# Compositional Data Analysis in Time-Use Epidemiology: What, Why, How

**DOI:** 10.3390/ijerph17072220

**Published:** 2020-03-26

**Authors:** Dorothea Dumuid, Željko Pedišić, Javier Palarea-Albaladejo, Josep Antoni Martín-Fernández, Karel Hron, Timothy Olds

**Affiliations:** 1 Alliance for Research in Exercise, Nutrition and Activity (ARENA), University of South Australia, Adelaide 5001, Australia; tim.olds@unisa.edu.au; 2 Institute for Health and Sport, Victoria University, Melbourne 3000, Australia; zeljko.pedisic@vu.edu.au; 3 Biomathematics and Statistics Scotland, EH9 3FD Edinburgh, Scotland, UK; javier.palarea@bioss.ac.uk; 4 Department of Computer Science, Applied Mathematics and Statistics, University of Girona, 17003 Girona, Spain; josepantoni.martin@udg.edu; 5 Department of Mathematical Analysis and Applications of Mathematics, Palacký University, 77146 Olomouc, Czech Republic; hronk@seznam.cz

**Keywords:** compositional data, physical activity, sedentary behavior, sleep

## Abstract

In recent years, the focus of activity behavior research has shifted away from univariate paradigms (e.g., physical activity, sedentary behavior and sleep) to a 24-h time-use paradigm that integrates all daily activity behaviors. Behaviors are analyzed relative to each other, rather than as individual entities. Compositional data analysis (CoDA) is increasingly used for the analysis of time-use data because it is intended for data that convey relative information. While CoDA has brought new understanding of how time use is associated with health, it has also raised challenges in how this methodology is applied, and how the findings are interpreted. In this paper we provide a brief overview of CoDA for time-use data, summarize current CoDA research in time-use epidemiology and discuss challenges and future directions. We use 24-h time-use diary data from Wave 6 of the Longitudinal Study of Australian Children (birth cohort, n = 3228, aged 10.9 ± 0.3 years) to demonstrate descriptive analyses of time-use compositions and how to explore the relationship between daily time use (sleep, sedentary behavior and physical activity) and a health outcome (in this example, adiposity). We illustrate how to comprehensively interpret the CoDA findings in a meaningful way.

## 1. Introduction: The Time-Use Epidemiology Framework

The most commonly studied time-use behaviors in relation to health are physical activity (PA), sleep and sedentary behavior (SB). Modern research on PA and health has a long history, with celebrated epidemiological studies dating back to the 1950s and 1960s [1]. It was not until the late 1990s and early 2000s that SB emerged as an “independent” risk factor. More recently still, PA epidemiology has joined forces with sleep epidemiology to cover all “movement behaviors” and take into consideration the time spent across the entire 24-h day.

Since PA, SB and sleep are mutually exclusive and exhaustive parts of the overall 24-h day, the time dedicated to one behavior can only be changed by simultaneously changing one or more of the other behaviors by the same net duration. This means that the observed health effects of changing one behavior (e.g., increasing PA time) will be partly due to compensatory changes in the others (i.e., decreasing SB and/or sleep). Activity behaviors and the time spent on them should not be analyzed or interpreted in isolation from the remaining behaviors, as all behaviors are necessarily related to each other. As stated by Shanahan and Flaherty, “time devoted to one domain of activity takes on full meaning only when viewed in terms of its functional relation to time spent in other domains” [2] (p. 386). This realization has catalyzed a shift away from exploring relationships between time spent in one behavior (e.g., PA) in isolation and some health outcome (e.g., dementia), to exploring how the intrinsic interplay and reallocations of time among daily behaviors are associated with health [3,4].

The conceptual framework for an integrated 24-h time-use epidemiology paradigm, rather than individual behavior paradigms, is now widely accepted [4,5,6,7,8,9]; recently, 24-h activity guidelines have been released in Canada [10], Australia [11], New Zealand [12], South Africa [13], Finland [14], Croatia [15] and by the World Health Organization [16]. However, changing how we think about time use has brought new challenges. In time-use epidemiology, statements like “get at least 30 min of moderate-to-vigorous PA (MVPA) five times per week” or “reduce time spent in SB” are in this sense incomplete, since there are no recommendations about how the rest of the day should be accommodated. In contrast, messages like “sit less, move more” (in spite of their vagueness) are consistent with the time-use epidemiology paradigm, as they are explicit appeals to swap one behavior for another (such as in the Australian *Swap It* campaign) [17].

This paper aims to describe the specific characteristics of time-use data, discuss a statistical approach (compositional data analysis, CoDA) increasingly used in the field and describe some of its benefits, challenges and limitations. Section 2 shows why time-use data convey relative information and what this implies for statistical analysis. Section 3 introduces CoDA as an approach suitable for time-use data and demonstrates the application of CoDA for descriptive analysis. Data from the Longitudinal Study of Australian Children (LSAC) are used as an example. In Section 4, CoDA for inferential analysis of time-use data against health outcomes is demonstrated using the same example dataset. Special emphasis is given to the interpretation of findings from CoDA regression models. Section 5 discusses difficulties and potential limitations of the CoDA approach. A concluding Section 6 summaries the current state of the field in regard to CoDA.

## 2. Time-Use Data Convey Relative Information

Daily time-use behaviors are co-dependent on each other and bounded to 24 h/day. The data therefore convey relative information rather than absolute information. The issue is that most standard statistical methods are not intended for the analysis of relative information. Instead, standard statistical methods are suited for absolute information. An absolute scale implies that, for example, a daily increase of one hour of PA represents the same variation whether one is initially doing one hour or 10 h of PA. That is, we assume that subtraction is the natural operation to measure the differences and variability. But, when we conceptualize time-use data as data carrying *relative* information, i.e., the relevant information is in the *ratios* between behaviors, an increase of one hour for someone with a baseline level of one hour is a 100% increase; while for someone with a baseline level of 10 h, it is only a 10% increase.

Most analyses of time-use data have used statistical methods which focus on the absolute information in the data. *“Leave-one-out” regression*—where one domain of time use, for example, SB or light PA (LPA), is omitted to avoid perfect multi-collinearity—has been commonly used [18]. Ordinary *partial least squares (PLS) regression* has been used with a focus on dealing with severe or perfect multicollinearity [19,20]. *Non-compositional isotemporal substitution* [21] has been widely applied to time-use research, originally for waking hours only, but more recently also for 24-h data [22]. Another approach to accommodate the 24-h day in an analysis involves not using cut-points to define activities by their intensity (e.g., sleep, SB and light, moderate and vigorous PA) and instead looking at daily activity as a function of energy expenditure against time [23]. Using *functional data analysis* [24], these functions are regressed against outcomes; interpretation in terms of energy expenditure bands can be retrospectively considered. All these “absolute” analytical strategies have advantages in the way data are modelled and results can be interpreted. However, they all share the same major disadvantage—they are not suited for time-use data which convey relative information.

CoDA takes a different approach. It respects the relative nature of time-use data by expressing the information as a set of logratios. The logratios are then analyzed in traditional statistical models instead of raw absolute values (e.g., minutes of MVPA). Although the main part of the analysis is done on logratios, a set of procedures is available to interpret results of CoDA back into the original terms (i.e., minutes/day).

## 3. The Rationale and Methods of CoDA

The basic principles and rationale for CoDA were formalized in 1982 by John Aitchison [25]. The approach was initially used mainly for the analysis of geochemical data, typically referring to chemical compositions, but it has since been implemented across a wide range of disciplines, including most recently molecular biology and microbiome studies [26,27]. CoDA is not only suited to data which are constrained to sum to a constant absolute amount (such as 24 h). Some compositional data have varying totals, for example, body composition data, which may capture kilograms of truncal fat, non-truncal fat and fat-free mass, sum to different total body masses for each participant, yet for analysis these data are commonly expressed as percentages. This also implies that the relevant information allowing for comparability between individuals is relative and should be analyzed using a CoDA approach. An example relevant to time-use data may be the composition of waking hours only (i.e., SB, LPA and MVPA). However, without loss of generality, this paper focuses on CoDA for 24-h time use. Interestingly, one of Aitchison’s first examples of the application of CoDA was to analyze a statistician’s daily time-use budget [28].

Time-use compositions are made up of *parts* (activity domains) that, in the case of daily time use, are always positive and sum to the same total. The total can be expressed as 24 h, 1440 min, 100%, 1 or any other number, provided the activity parts are scaled accordingly. The property called “scale invariance” is one of the fundamental principles of the CoDA approach: the results of analysis are the same regardless of the scale used [28]. Because the total amount is the same for everybody, the absolute values of the total and the absolute amounts of time spent in behaviors are irrelevant for the analysis—they carry no additional information beyond the proportions of time spent in different behaviors; behaviors are only meaningful in relation to each other. The possible values any time-use data point can take are constrained to fall within certain limits, i.e., the time-use data’s sample space. The sample space for time-use compositions is called the “Simplex”, whereas the usual reference sample space for absolute data is the “real space”. The data’s sample space defines the data’s geometry and which mathematical operations (e.g., addition, multiplication) can be applied to the data.

**Figure 1:**
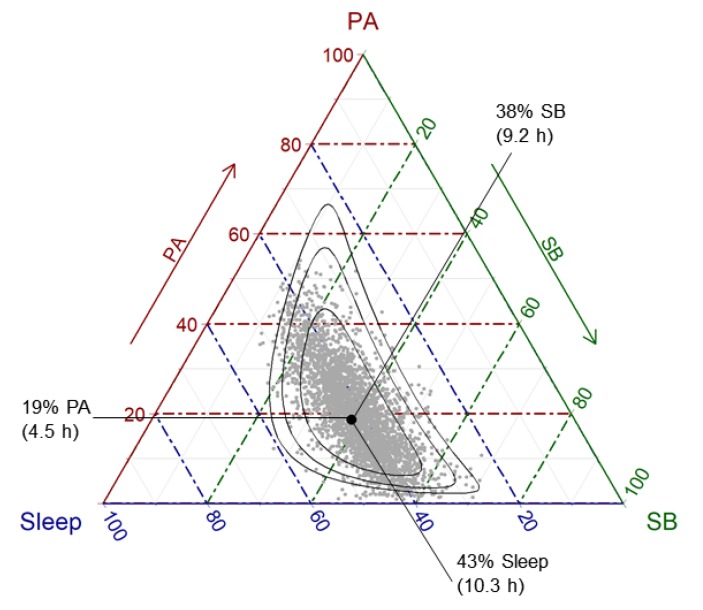
The ternary diagram: The Simplex sample space for a three-part composition is a triangle. Time-use data are from the Longitudinal Study of Australian Children, birth cohort, Wave 6. Black dot represents the compositional center of the time-use dataset, surrounded by 75%, 95% and 99% predictive regions from fitting a logratio normal distribution, which reflects the relative scale of compositional data [31] (see Section 3.1 for further details). PA = physical activity; SB = sedentary behavior.

Consider the sample space for a three-part 24-h time-use composition, dividing the day into three mutually exclusive and exhaustive time-use behaviors (e.g., PA, SB and sleeping time). The Simplex sample space in this case is a triangle as shown in the plot below (Figure 1), called a ternary diagram. Figure 1 shows the time-use compositions of 3228 Australian children aged 10.9 ± 0.3 y. The time-use compositions were derived from 24-h time-use diaries collected during the sixth biennial wave of LSAC (birth cohort, 2013–2014) [29,30]. A parent provided written informed consent for their child’s participation in LSAC. The study was conducted in accordance with the Declaration of Helsinki and ethical approval was gained from the Australian Institute of Family Studies Ethics Committee, which is a Human Research Ethics Committee registered with the National Health and Medical Research Council (Australia). LSAC also gathered participants’ height and weight, measured by trained researchers using a portable rigid stadiometer and glass bathroom scales. Throughout this paper we use the time-use dairy data as an example of a three-part 24-h time-use composition. We focus on the application of CoDA rather than the methodological details of the sample dataset, however details of LSAC study design and the collection of time-use diaries and measures of height and weight (for BMI) are provided in the Supplementary Materials. 

In Figure 1, it can be seen that any possible daily mix of PA, SB and sleep must lie somewhere on the ternary plot and cannot be found anywhere outside of the triangle. A data-point falling outside this triangle would denote impossibilities: either negative time—or more than 24 h in one day.

When non-compositional statistical models are applied to time-use data, it is incorrectly assumed that the time-use data can exist anywhere in real space (i.e., outside the Simplex). For example, predictive regions of a typical Gaussian distribution would be outside the ternary diagram in Figure 1. Non-compositional methods also assume that mathematical operations for real space (e.g., addition and multiplication) are compatible with the natural geometry of the data. However, mathematical operations are defined differently in the Simplex compared to real space. For example, the basic operations of addition and multiplication in real space are redefined as the equivalent operations of perturbation and powering in the Simplex space [28]. These differences have implications for the analysis of compositional data.

### 3.1. The Descriptive Analysis of Compositional Data

The *center* or *compositional mean* of a 24-h compositional data set is expressed as a vector of geometric means of its parts, rescaled (without loss of information) to sum to 24 h (or 1440 min). In Figure 1, the compositional mean of the sample is depicted by a black dot. The geometric means of the parts can equivalently be expressed in percentages (Sleep; SB; PA) = (43; 38; 19) or h/d (10.3; 9.2; 4.5) or min/d (617; 553; 269). The relative difference between the individual parts remains identical, e.g., PA/Sleep = 19/43 = 4.5/10.3 = 269/617 = 0.43.

In statistics it is accepted that the geometric mean is the natural central tendency statistic for data varying in a relative scale, typically exhibiting an asymmetric distribution and subject to multiplicative changes [32]. The individual distribution of a time-use variable (e.g., graphed using a histogram) commonly shows such an asymmetric profile, with, for example, values at the lower range of MVPA being much more frequent than higher values. It has been mathematically proven that the geometric means provide the optimal estimator of the population means for compositional data [32]. This is intuitive, because it effectively reflects the *relative scale* of time-use data: components with small values naturally induce more relative variability. For example, increasing MVPA by 10 min might double daily MVPA time, while doing the same for SB would result in a negligible relative increase. Accordingly, small values contribute greatly to the variability of relative data. The arithmetic mean minimizes the *absolute* distance between datapoints (i.e., as measured by a standard ruler, also called “Euclidean distance”), while the compositional mean minimizes the *relative* distance (also called “Aitchison distance”) between datapoints.

Thinking in terms of compositional distance is not only important for finding the center of compositional datasets but underpins many other statistical procedures. For example, cluster analysis and latent profile analysis rely on distance measures to detect homogenous groups in the population. Distance measures are required for MANOVAs to test for differences between compositional means. Regression analyses aim to minimize the distance between compositional residuals and the regression line.

**Figure 2:**
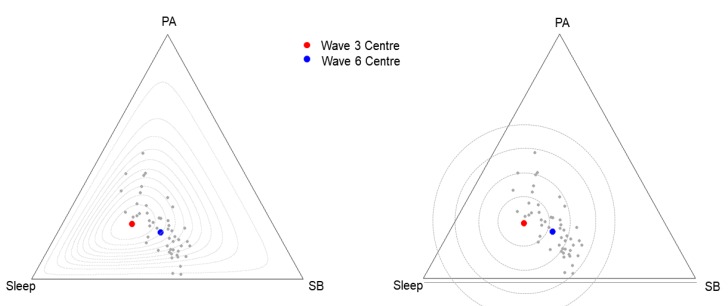
Evenly spaced distance contours around the center of the Longitudinal Study of Australian Children Wave 3 birth cohort time-use compositions (red). The left panel shows contours defined by relative (Aitchison) distance; the right panel shows contours defined by absolute distance (Euclidean). The center of Wave 6 time-use composition is shown in blue. Grey dots represent data from 50 randomly sampled Wave 6 participants. PA = physical activity; SB = sedentary behavior.

Figure 2 compares relative and absolute measures of distance. The compositional centers of Wave 3 time-use compositions (red) and Wave 6 time-use compositions (blue) of children with valid data at both LSAC waves (n = 2881) are shown. In addition, the time-use compositions of 50 randomly sampled children from Wave 6 are shown as grey datapoints. During the six years from Wave 3 to Wave 6, children increased their SB; the reallocated time came mostly from sleep and a little from PA. To quantify potential divergence from the Wave 3 time-use composition, we created an index of change, normalized to a range of [0,1], based on Aitchison distance.

The plot on the left has change contours evenly spaced at 0.1 (10%) increments from the Wave 3 center, using relative (Aitchison) distance. The contour lines stay within the confines of the data’s sample space. In contrast, the plot on the right has change contours spaced by 10 percentage units but considering them in an absolute change scale (Euclidean distance). The contours extend beyond the triangle (i.e., beyond the possible values any composition can take), because the distance measure is not compatible with the Simplex sample space.

Consider a hypothetical situation where the Wave 3 time-use compositions of all 50 randomly sampled children were identical to the Wave 3 center. We could use the change contour lines to explore how much their time-use composition has changed by Wave 6. The shape of circles in the Euclidean distance plot (right) would suggest that an increase or a decrease is symmetrical. In addition, the equal separation between the circles along the Simplex suggests that an increase of one hour in any part represents the same variation regardless of the location. In contrast, consistent with the relative nature of time-use data, the iso-contours in the Aitchison distance plot (left) are not symmetrical in all directions and the separation diminishes when one approaches an edge or a vertex of the ternary diagram.

The variability of a compositional dataset is not described using univariate measures such as the standard deviation of an individual part, because as one part varies, one or more other parts must also vary. Instead, a variation matrix [28] is used to describe pair-wise variation between parts. The variation matrix of the LSAC Wave 6 time-use compositions is presented in Table 1 (upper triangle). Each value is the variation in the logratio of parts. Highest variation is observed in the logratio of PA vs SB. The spread of the data points (Figure 1) also suggests that most of the variation is between PA and SB. The data cloud appears almost parallel to the grid lines for sleep (broken blue lines). This suggests that as PA levels increase within the sample, SB tends to decrease to compensate (and vice versa), while sleep stays fairly constant.

The lower triangle of the variation array (Table 1) displays the mean of the pairwise logratios. The values are all negative, meaning the logratio denominators are on average larger than the numerators. For example, the average of ln(PA/Sleep) is −0.83. From this we can say that PA duration is on average shorter than Sleep duration. We can calculate how much shorter it is by computing exp(−0.83) = 0.43. Multiplying 0.43 with the center of Sleep (617 min/d) gives 269 (min/d), the center of PA.

The heart of the CoDA methodology is to express the time-use data in relative terms, as a set of logratios, which are simply the logs of ratios of time-use parts [e.g., the normalized log of the ratio of PA to the remaining parts of the composition (SB and sleep)]. The logratios form vectors in real space, which can then be used to represent the time-use data in typical statistical models [33].

**Figure 3:**
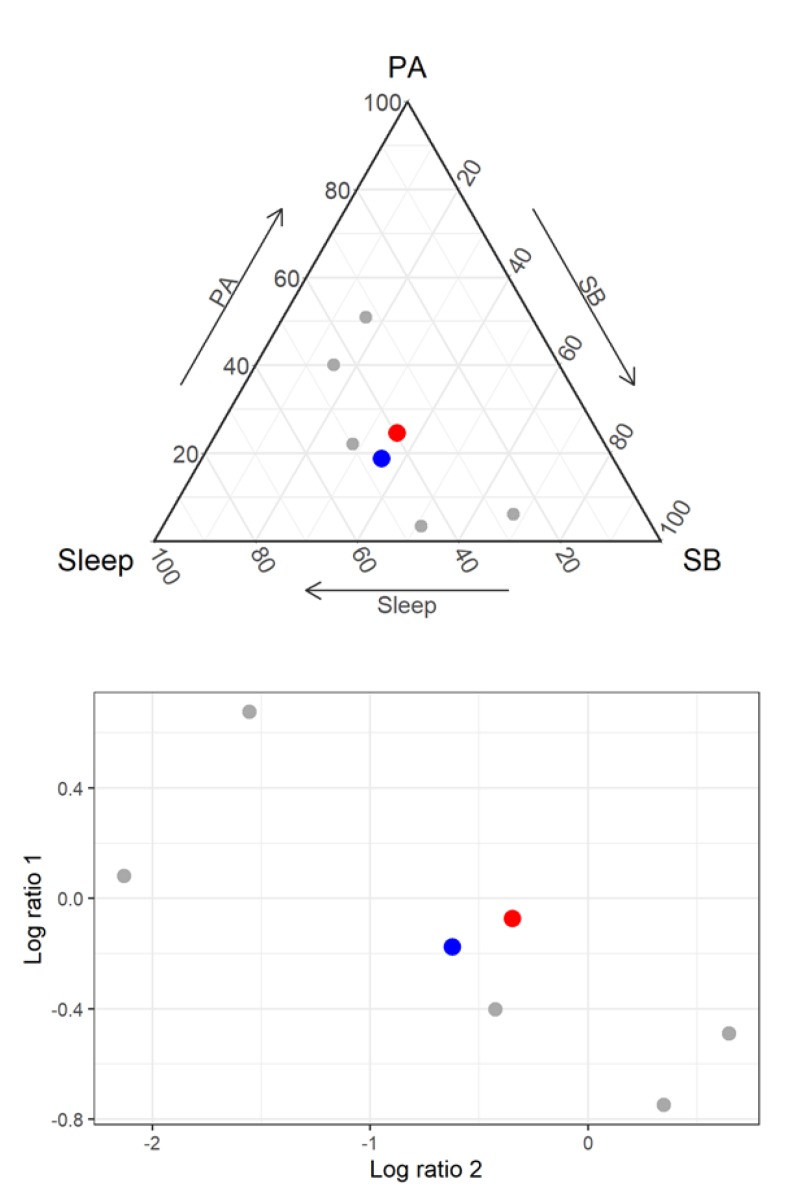
Five randomly selected time-use compositions from the Longitudinal Study of Australian Children (birth cohort, Wave 6). Top panel shows the compositional mean (blue) and arithmetic mean (red) in the ternary diagram. Bottom panel shows real space isometric logratio representation, with their compositional mean (blue) and arithmetic mean (red). PA = physical activity; SB = sedentary behavior.

Figure 3 (top panel) shows both the compositional mean (relative scale) and the arithmetic mean (absolute scale) of time-use compositions of five randomly sampled children from the LSAC study. Figure 3 (bottom panel) shows a real space logratio representation of the five compositions. The compositional mean (blue) can be seen to provide a better representation of the center of the datapoints compared to the arithmetic mean (red).

Formulas to create the logratios are readily available [3,33,34,35,36]. A number of logratios have been defined, including additive, centered and isometric logratios, each with advantages and disadvantages depending on their intended use [35]. CoDA studies have typically used the R [37] free software system for statistical computing because R has several established packages specifically designed for CoDA [38,39,40]. The basic formulas for CoDA can be implemented in many commonly used statistical programs to create logratios as new variables for use in statistical analyses. The OpenCoDa website (https://opencoda.net) offers online CoDA resources and friendly point-and-click web apps based on R to conduct common analyses, particularly tailored to the needs of time-use epidemiologists and movement-behavior researchers. CoDaPack [41] is a user-friendly, stand-alone and multi-platform package implementing CoDA functions, which resembles the look and feel of popular statistical packages with graphical user interface.

## 4. Understanding the Results of CoDA Studies

To date, CoDA has been used to explore the cross-sectional and longitudinal associations between time use and health [42]. Most studies have considered a four-part activity behavior composition (sleep, SB, LPA and MVPA), usually derived from accelerometry. However, in this paper we demonstrate the CoDA approach using a three-part composition (sleep, SB, PA) because it is easy to visualize in two dimensions.

### Compositional Regression Analysis

The time-use composition (expressed as set of logratios) has been modelled as the dependent variable in multivariate linear regression analyses [43,44,45]. Most commonly, however, researchers have used regression analysis with the time-use logratios as the explanatory variables and a health outcome as the dependent variable.

**Figure 4:**
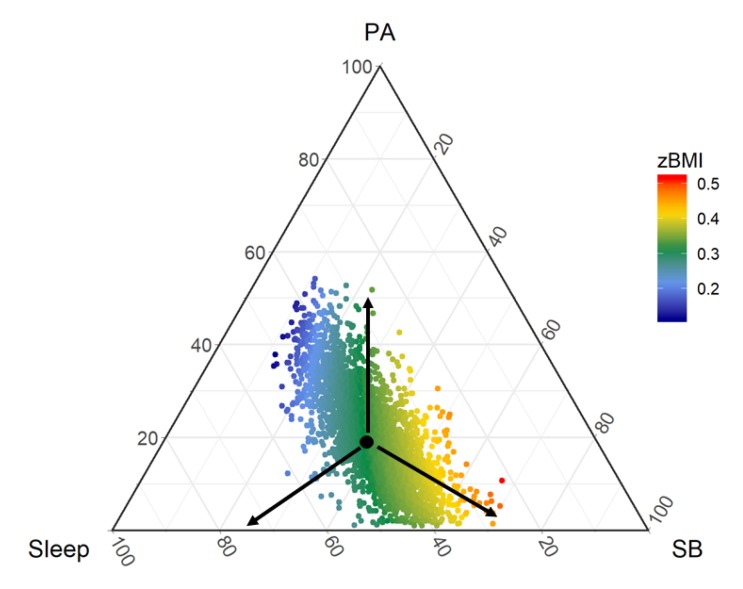
Estimated zBMI response surface. Arrows indicate reallocation of time to the part in the corner, taking it equally from the remaining parts. PA = physical activity; SB = sedentary behavior; zBMI = estimated body mass index z-score.

Regression analyses have typically used isometric logratio coordinates [46] as these enable meaningful interpretation of individual regression coefficients [47]. The word “coordinates” refers to a more generic mathematical concept of the resulting logratios being points/coordinates in the real space. The datapoints in Figure 4 are coordinates in real space. A particular, useful isometric logratio representation, called *pivot coordinates* [48] isolates (in one of the coordinates) the contribution of a specific part of the time-use composition relative to the remaining parts. Such a coordinate can sequentially be created for each part of the composition. Pivot coordinates of time-use compositions have been widely used as explanatory variables in multivariate linear regression models.

The pivot coordinate for MVPA has emerged as having the strongest beneficial associations with a range of health indicators in many adult and pediatric populations [49,50,51,52,53,54]. Interestingly, the pivot coordinate for LPA has frequently appeared to be unfavorably associated with health outcomes in children and adults [3,49,55,56], although it has been beneficially associated with health in older adults [57]. Some studies report unfavorable associations between the pivot coordinate for SB and outcomes [3,50,55,58]. The pivot coordinate for sleep duration has generally been positively associated with health outcomes [35,55,59]. CoDA typically yields asymmetrical dose-response curves. For example, the benefits of incrementally increasing MVPA relative to the other behaviors appear to diminish, whereas the adverse effects of incrementally decreasing MVPA relative to the other behaviors appear to escalate.

It is important to be clear what these results mean. Unlike earlier analytical approaches, results from CoDA are not meant to be interpreted univariately, in coherence with the particular nature of time-use data [2]. One must always consider changes in behaviors relative to other behaviors. For example, one CoDA analysis reported an apparently counter-intuitive positive association between moderate-intensity PA and cardiometabolic risk [60]. However, in this instance, the association was actually between moderate-intensity PA (MPA), relative to the geometric mean of the remaining day [which included vigorous-intensity PA (VPA)] and cardiometabolic risk. The positive coefficient of that pivot coordinate is reflecting the higher metabolic risk of the net time reallocation, that is, it includes the relative reduction of VPA and SB. MPA *per se* is therefore not associated with greater cardiovascular risk, but its *net* reallocation (relative to VPA and SB) in this population is. This difficulty could be addressed by analyzing the association of other general logratios with cardiometabolic risk [35,47,61]. In addition, thinking in terms of time *reallocations* is helpful when interpreting CoDA results.

To illustrate CoDA regression and its interpretation, we explore the relationship between three-part time-use composition and measured body mass index z-score (zBMI) from Wave 6 of LSAC, birth cohort. Three sets of pivot coordinates were regressed against zBMI, adjusted for age, sex and socioeconomic position (a composite z-score derived from family level factors, such as parental education, employment and income). The time-use composition was associated with adiposity (F = 4.0, *p* = 0.02).

The beta estimates for the pivot coordinates (Table 2) suggest that as sleep increases (and SB and PA decrease), zBMI decreases. As SB increases (and PA and sleep decrease), zBMI increases. There appears to be no relationship between PA, relative to the remaining behaviors and zBMI.

Color-coding the datapoints according to their model-predicted zBMI (red = high zBMI, blue = low zBMI) shows that the steepest gradient is in the direction of SB, towards the bottom right corner of the triangle (Figure 4). The black arrow towards SB reflects the relationship given by the pivot coordinate estimate for SB vs Remaining, i.e., as SB increases and the remaining behaviors (PA and sleep) decrease in equal proportions, there is a steep incline in estimated zBMI. The zBMI response for increasing PA while equally reducing SB and sleep (vertical arrow towards PA) is flat, there is no variation in color. However, overall, there is a beneficial gradient between PA and zBMI, because lower zBMI (blue) is observed at higher proportions of PA (towards the peak of the triangle) and higher zBMI (red) at lower proportions of PA (base of the triangle). In other words, if time is reallocated to PA from SB (without changing sleep), the estimated response is beneficial. In this case, interpreting the pivot coordinate for PA in a univariate sense (i.e., to represent PA) would lead to misleading or incomplete conclusions.

## 5. Challenges for CoDA

CoDA is increasingly widely used in time-use studies, but as with every new paradigm, there are challenges and critiques.

### 5.1. Zero Values

A limitation of CoDA is that zero values cannot be included in logratios, because dividing by zero or taking the logarithm of zero are undefined mathematical operations [62,63,64,65]. Zero values in time-use CoDA datasets have been classified as either *rounded* zeros or *essential* zeros. Mostly, time-use zeros are considered as rounded because it can usually be assumed that an individual would accumulate some amount of time in the behavior if the measurement took place over a long enough period or if it was done using a sensitive enough measurement tool [62,65]. For the commonly analyzed time-use composition (i.e., the one consisting of sleep, SB, LPA and MVPA) and for most study populations, the occurrence of zeros is minor relative to the size of the dataset and they can indeed be considered as rounded zeros. In such cases, a popular approach is to rely on statistical imputation methods [63], that is, replacing zeros with sensible small values based on the available information. Unlike rounded zeros, essential zeros are considered true representations of the underlying reality (e.g., time spent walking for a wheelchair bound individual). Although some strategies have been proposed to deal with essential zeros in particular settings, there is no general “plug-in” approach to tackle the problem [63,66]. Future work in the field may explore some potential strategies, such as stratifying by subcompositions according to zero patterns [66] or considering an approach that tries to deal indirectly with them [67]. The R package zCompositions [40] includes some tools to assist in this decision. One pragmatic strategy which often helps to circumvent the issue is to merge (amalgamate) two or more time-use components (e.g., MPA and VPA) into a single variable in a meaningful way, before conducting the analysis.

### 5.2. Multicollinearity

One of the great advantages of CoDA is that it eliminates the perfect multicollinearity that arises when raw data of all time-use domains are entered into the same model. However, as could be the case for any kind of data, the logratios may still be highly collinear, which has been a matter of concern to some authors [60]. It is important to note that CoDA only avoids perfect multicollinearity among predictors when isometric or additive logratios are used to represent the composition. By contrast, the centered logratios are perfectly multicollinear by design. The centered logratios have utility for certain analyses but should not be used in linear regression models or to explore the data’s correlation structure [68,69].

When isometric logratios are used as predictors in regression models, individual regression coefficients can be interpreted in a meaningful way. However, as is the case with any multiple linear regression model, high collinearity between predictors may affect the stability of regression coefficients and the results of significance tests [70]. If interpretation of individual regression coefficients is required and high multicollinearity between logratios is an issue, it may be appropriate to explore extensions of regression methods that are designed for highly collinear explanatory variables. For example, *Partial least squares regression (PLSR)* is a well-known method to deal with highly correlated explanatory variables; it has been used to model movement behavior variables in relation to health outcomes [19,20,60]. However, to be congruent with the relative nature of time-use data, PLSR should be applied to logratio coordinates [71,72].

It is well-known that multicollinearity leads to numerical instability and inflation of the variability of the regression coefficients for highly correlated individual predictors; hence inflating their standard errors, reducing the power of the associated significance tests and making it complicated to disentangle individual effects. However, it does not affect predictions made on the same or new data having analogous degree of collinearity as the data used to fit the model [70,73]. Compositional isotemporal substitution [34] uses model-based prediction to provide a way of interpreting compositional regression models in an intuitive, meaningful way. Estimates are derived for re-allocating time between the activity behaviors.

**Figure 5:**
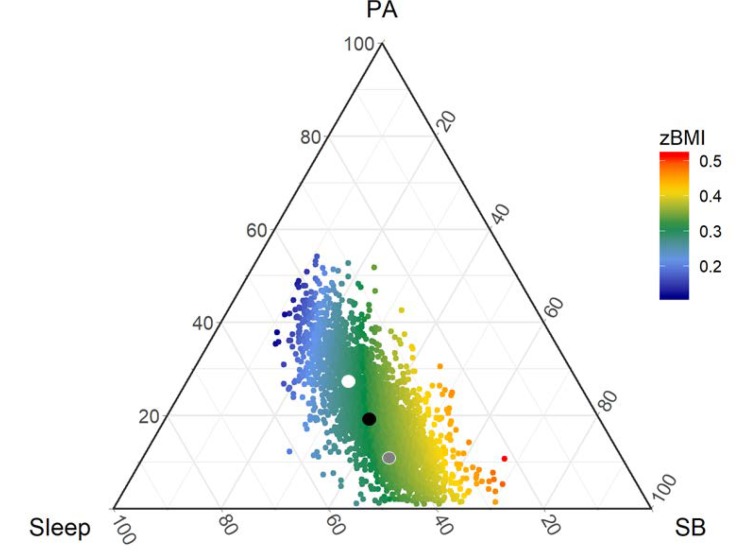
Isotemporal substitution of 2 h. Reallocation to PA from SB (white dot) and to SB from PA (grey dot), starting from the compositional mean (black dot). Data are from the Longitudinal Study of Australian Children, Wave 6, birth cohort. All analyses adjusted for sex, age and socioeconomic position. PA = physical activity; SB = sedentary behavior, zBMI = estimated body mass index z-score.

Using the Wave 6 LSAC time-use data, we observe the estimated difference in zBMI when two hours (8.2% of 24 h) are reallocated from SB to PA, keeping sleep constant (Figure 5). The time reallocation is made to the compositional center (black dot), resulting in a new composition (white dot), where zBMI is estimated to be lower than at the compositional center (-0.03, 95% CI: -0.06; -0.01). A reallocation of the same absolute duration of time, but in the opposite direction (i.e., taking 2 h from PA and giving it to SB while keeping sleep constant, shown by the grey dot in Figure 5), is equivalent only in an absolute sense (i.e., using Euclidean distance), but not in a relative sense. Thus, estimated differences in outcomes may not be symmetrical when the same absolute duration is added or taken away from a part. The estimated difference in zBMI for reallocating 2 h to SB from PA (black dot to grey dot) is +0.02 [−0.01;0.06].

Reallocations of time could also be considered from all other components pro-rata-to-one (*one-for-remaining reallocation*, e.g., from sleep, SB and LPA to MVPA). Other types of reallocation, including *empirical* reallocation (the way people do in fact reallocate time) are possible.

Because reallocation approaches rely on the predictive power of the overall model, multicollinearity is less of an issue. However, there is a problem with predicting outcomes for compositions near the fringes of the Simplex where components are very close to zero or 100% because predictions will approach +/− infinity. Future work may explore iterative algorithms for making predictions for compositions with parts that are close to zero.

### 5.3. Non-Linearity

It has been said that “composition analysis assumes nonlinear relationships” [8] (p. 460), but this misconception arises because CoDA works with logratios. When linear algebra is performed on logratios and results are anti-logged, relationships appear non-linear in real space. As discussed in Section 5.2 (Figure 5), dose-response relationships between activity domains (relative to the other parts of the composition) will generally appear asymptotic or logarithmic. Indeed, when one uses CoDA, one assumes a Simplex geometry where *multiplicative* error is the natural *change*, which is linear in the logarithmic scale. A standard non-compositional approach, however, will result in symmetrical straight lines, unless transformations such as power transformations are used.

**Figure 6:**
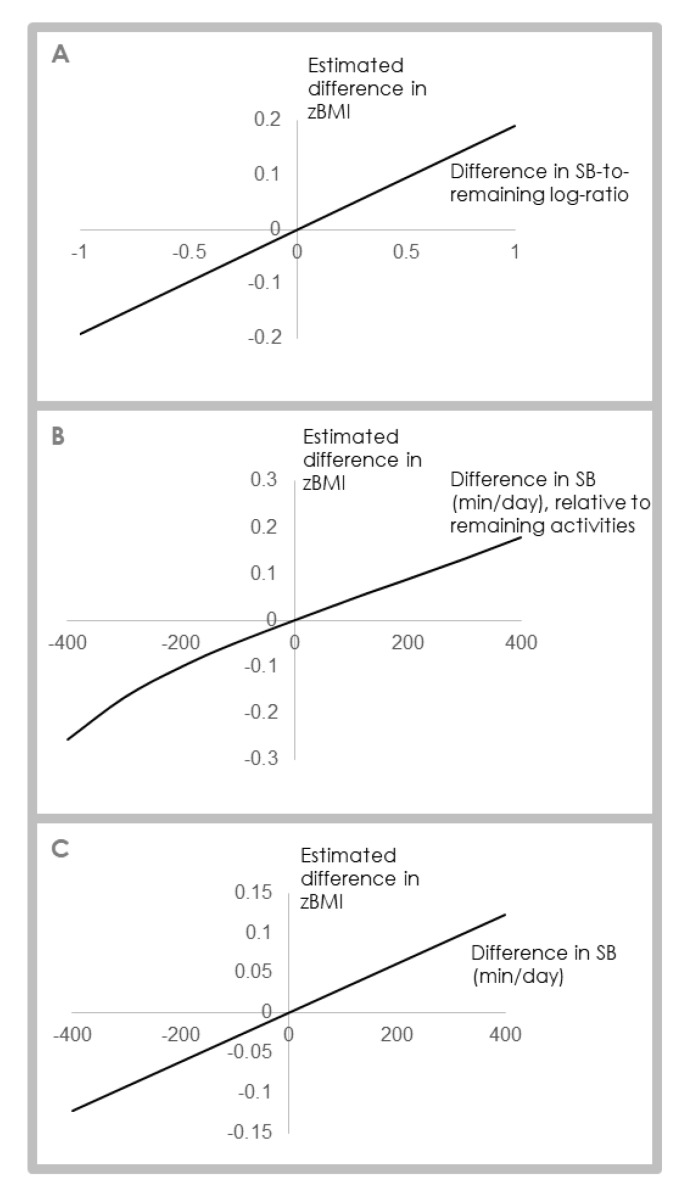
Relationship between daily activity behaviors (specifically SB) and zBMI. Panel A: Difference in zBMI associated with difference in SB-to-remaining activities expressed as a pivot coordinate, as estimated by compositional linear regression. Panel B: Difference in zBMI associated with difference in SB-to-remaining activities expressed in min/d, as estimated by compositional linear regression. Panel C: Difference in zBMI associated with difference in SB (min/d), as estimated by linear regression. Data are from the Longitudinal Study of Australian Children, Wave 6, birth cohort. All analyses adjusted for sex, age and socioeconomic position. zBMI = body mass index z-score; SB = sedentary behavior.

Figure 6 shows estimated zBMI response-curve shapes derived from multiple linear regression models for a three-part time-use composition (sleep, SB and PA). Data are from the LSAC birth cohort, Wave 6. Panel A displays results from a compositional linear model with pivot coordinates as explanatory variables. It shows a linear relationship between the ratio of SB, relative to the remaining activities (sleep, PA) and zBMI. Panel B displays results from the same compositional linear model as A, however the estimates have been back-transformed into minutes/day of SB, relative to the remaining activities. The response curve in Panel B appears non-linear at its lower end, but it is linear in its original log-scale (panel A). Panel C shows the linear relationship estimated from a typical linear regression model with raw values of SB (min/day) as the predictor.

Every modelling approach has limitations about how it can represent the world; it can only ever be an approximation of reality. For example, if we only use linear models, we can only represent reality using straight lines. If we use a quadratic function, we can fit U-shaped and inverted-U-shaped patterns. In the same way, different parts of the logarithmic curve can provide satisfactory descriptions of rising and falling linear and non-linear associations. CoDA is a useful methodology whose representations of the shape of the relationship are congruent with the nature of the sample space of time-use data.

## 6. Conclusions

CoDA is not linked to any particular method, it is a *methodology* for dealing with compositional data, such as time-use data. The use of CoDA in time-use epidemiology is not without its challenges. They arise as the field is shifting the focus and interpretations of findings from a “univariate” approach to a multivariate, integrated conceptualization of time use. Despite the challenges, CoDA currently provides us with the ability to comprehensively analyze and interpret the integrated relationships between time-use data and health.

This paper demonstrated the application of the CoDA approach on time-use diaries from LSAC. When interpreting and generalizing the findings presented, it is important to consider the strengths and weaknesses of the study design. Strengths include the large, nationally representative cohort of Australian children. However, there are also some weaknesses that need to be acknowledged. First, time-use data were self-reported by children aged 10–11 years. Children of this age have been shown to provide acceptable estimates of their time use [74]; the interviewer-assisted approach employed in the study will have improved the quality of the data. Nonetheless, the potential for social desirability and recall bias remains. In addition, only one day was sampled which may not be representative of the child’s habitual time use. We did not account for the fact that children’s time use may differ across seasons, weekend days/weekdays and school terms/holidays. It should also be remembered that the regression analyses between time use and adiposity were performed on cross-sectional data, therefore the direction of the relationship cannot be established.

The field of time-use epidemiology has adopted CoDA relatively quickly. To date the CoDA approach has primarily been applied to create linear regression models. Future possibilities include embedding the CoDA approach into other types of analyses that may be relevant for time-use epidemiology, such as mediation analyses, causal models, time-series analyses and optimization. Future studies should continue developing new CoDA methods to facilitate research in time-use epidemiology.

## Figures and Tables

**Table 1:** Variation array of three-part time-use composition.

		Mean Variation of the Pairwise Logratio			Center
		Sleep	SB	PA	(min/d)
Numerator of logratio	Sleep		0.13	0.39	617.5
	SB	−0.11		0.78	553.1
	PA	−0.83	−0.72		269.4
		Mean of the pairwise logratio			

The upper triangle is the variation of the logratios, for example, 0.13 is the average variation in ln(Sleep/SB). Note, 0.13 is also the average variation of the inverted logratio ln(SB/Sleep), i.e., the variation matrix is symmetrical. The lower triangle of the variation matrix is not shown. Instead, displayed in the lower triangle is the mean of the logratio, for example, −0.11 is the mean of ln(SB/Sleep). The means of the inverted logratios, i.e., ln(Sleep/SB) (not shown) are the inverse (i.e., mean ln(Sleep/SB = 0.11)). Compositional center of the dataset is shown in the final column. Data are from the Longitudinal Study of Australian Children time-use dairies (Wave 6, birth cohort). SB = sedentary behavior; PA = physical activity.

**Table 2:** Regression of pivot coordinates against body mass index z-score among n = 3228 children, Wave 6 LSAC birth cohort.

Pivot	Estimate	SE	t	p
Sleep vs Remaining	−0.21	0.11	−1.96	0.045
SB vs Remaining	0.19	0.07	2.56	0.010
PA vs Remaining	0.02	0.05	0.37	0.708

Analysis adjusted for sex, age and family level socioeconomic position. LSAC = Longitudinal Study of Australian Children; Remaining = remaining behaviors; SB = sedentary behavior; PA = physical activity.

## Supplementary Materials

The following are available online at https://www.mdpi.com/1660-4601/17/7/2220/s1.
