# Supplementary material for: Compositional Data Analysis in Time-Use Epidemiology: What, Why, How
Source: Int J Environ Res Public Health. 2020 Mar 26;17(7):2220. doi: 10.3390/ijerph17072220 (PMC7177981; doi:10.3390/ijerph17072220)
Supplement: Supplementary file 1 [file ijerph-17-02220-s001.pdf]

Supplementary File: The Longitudinal Study of Australian Children

The Longitudinal Study of Australian Children (LSAC), also known as Growing Up in Australia, is a nationally-representative longitudinal study initiated in 2003. The study is funded by the Australian Government and conducted in partnership between the Australian Government Department of Social Services, the Australian Institute of Family Studies and the Australian Bureau of Statistics, with advice provided by a consortium of leading researchers.

The sample was selected from the Medicare enrolment database held by the Health Insurance Commission. Children of the appropriate ages were selected and sent an 'invitation to participate' letter to the Medicare cardholder, along with a brochure about the study. Families were given four weeks to withdraw from the study.

There are two cohorts. A birth cohort of 5112 children aged 3-15 months at the start of the study and a child cohort of 4991 children aged 4-5 years at the start of the study. The cohorts are followed up every two years.

In the main paper, we use data from Wave 6 of the birth cohort (2013-2014), when the children were 10-11 years old. Data from Wave 3 (2007-2008) were also referenced.

Time-use diaries: Wave 6 Birth Cohort

In Wave 6, time-diaries were completed by the child participants [1]. The diaries captured the previous day, and only one day was recorded for each participant. The recorded day could be either weekday, weekend-day, school day or holiday.

Children were provided a paper-based diary (see Figure 1) and a pen with a digital clock. They were asked to record what they did throughout the day, and at what time they did it. After children completed their diary, they were visited by a research assistant (interviewer), who talked the child through the diary and attempted to fill in any missing information.

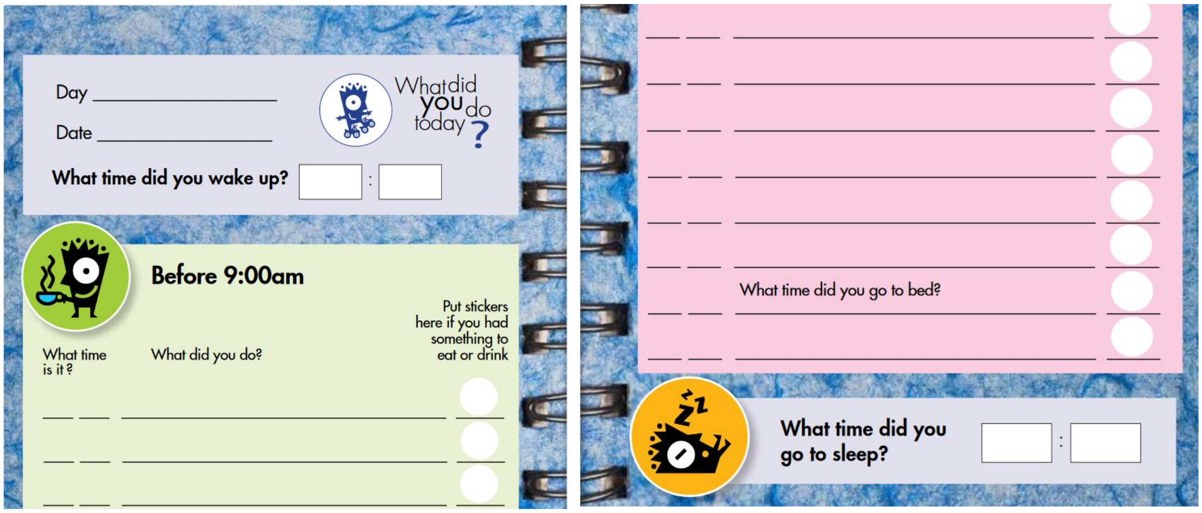

Figure 1. Excerpts from paper-based time-use diary used in Wave 6 of the Longitudinal Study of Australian Children, Birth cohort.

The research assistant coded the child's recorded activities into activity codes (Table 1). For the purposes of the example in the main paper, we further collapsed these activity codes into three categories – Sleep, sedentary behavior and physical activity, as shown in Table 1. Children were considered to have valid data if they had >288 minutes and <864 minutes sleep, >144 minutes sedentary behavior and >10 minutes physical activity.

Table 1. Classification of Wave 6 Birth Cohort Activity Codes into Physical Activity, Sedentary Behaviour and Sleep.

**Activity Code**	**Classification**	**Activity Code**	**Classification**
Attend courses excluding school university	Sedentary Behaviour	Home maintenance	Physical Activity
Attendance at concert theatre	Sedentary Behaviour	Hospitality including fast food	Physical Activity
Attendance at movies cinema	Sedentary Behaviour	Household management other	Physical Activity
Attending live sporting events	Sedentary Behaviour	Laundry clothes care	Physical Activity
By private motor vehicle bike	Sedentary Behaviour	Packing	Physical Activity
By public chartered transport	Sedentary Behaviour	Personal care medical health care	Physical Activity
Clerical office	Sedentary Behaviour	Personal care	Physical Activity
Communication	Sedentary Behaviour	Playing musical instruments or singing for leisure	Physical Activity
Creating maintaining websites	Sedentary Behaviour	Pool care chores	Physical Activity
Dentist orthodontist	Sedentary Behaviour	Private music lessons practice academic tutoring	Physical Activity
Doctor	Sedentary Behaviour	Purchasing administrative services	Physical Activity
Doing homework electronic device	Sedentary Behaviour	Purchasing consumer goods	Physical Activity
Doing homework not via electronic devices	Sedentary Behaviour	Purchasing other services	Physical Activity
Doing nothing	Sedentary Behaviour	Purchasing personal care services	Physical Activity

Downloading posting media	Sedentary Behaviour	Rubbish recycling	Physical Activity
Eating drinking	Sedentary Behaviour	Selling disposing of household assets	Physical Activity
Electronic device use	Sedentary Behaviour	Shopping	Physical Activity
Filling out the diary	Sedentary Behaviour	Showering bathing	Physical Activity
General application use	Sedentary Behaviour	Taking care of siblings chores	Physical Activity
General internet browsing	Sedentary Behaviour	Talking face to face	Physical Activity
Getting dressed getting ready	Sedentary Behaviour	Unstructured non active play	Physical Activity
Internet shopping	Sedentary Behaviour	Volunteering work	Physical Activity
Listening to music	Sedentary Behaviour	Walking pets playing with pets	Physical Activity
Medical health care	Sedentary Behaviour	Window-shopping	Physical Activity
Negative face to face communication	Sedentary Behaviour	Work other	Physical Activity
Non active activities	Sedentary Behaviour	Working in a family business or farm	Physical Activity
Non-verbal interaction	Sedentary Behaviour	Active activities	Physical Activity
Nothing	Sedentary Behaviour	Active club activities	Physical Activity
Online chatting instant messaging	Sedentary Behaviour	Archery shooting sports	Physical Activity
Physiotherapist chiropractor	Sedentary Behaviour	Archery shooting sports individual	Physical Activity
Playing games electronic device	Sedentary Behaviour	Archery shooting sports unstructured	Physical Activity
Playing games electronic device nod	Sedentary Behaviour	Athletics gymnastics	Physical Activity

Reading or being read to for leisure	Sedentary Behaviour	Athletics gymnastics individual	Physical Activity
Religious groups	Sedentary Behaviour	Athletics gymnastics unstructured	Physical Activity
Religious practice	Sedentary Behaviour	Ball sports	Physical Activity
Religious activities ritual ceremonies	Sedentary Behaviour	Ball sports individual	Physical Activity
School lessons	Sedentary Behaviour	Ball sports unstructured	Physical Activity
Spending time on social networking sites	Sedentary Behaviour	By foot	Physical Activity
Talking on a landline phone	Sedentary Behaviour	By bike scooter skateboard etc.	Physical Activity
Talking on a mobile phone	Sedentary Behaviour	Car boat bike care	Physical Activity
Texting emailing	Sedentary Behaviour	Car washing work	Physical Activity
Travel	Sedentary Behaviour	Cleaning grounds garage shed outside of house chores	Physical Activity
Video chatting	Sedentary Behaviour	Cleaning tidying	Physical Activity
Watching TV programs or movies videos	Sedentary Behaviour	Fitness gym exercise	Physical Activity
Weddings funerals rites of passage	Sedentary Behaviour	Fitness gym exercise individual	Physical Activity
Animal care	Physical Activity	Fitness gym exercise unstructured	Physical Activity
Animal care work	Physical Activity	Gardening lawn mowing	Physical Activity
Arts	Physical Activity	Gardening maintenance chores	Physical Activity
Attendance at museum exhibition art gallery	Physical Activity	Labourers and related workers	Physical Activity
Attendance at other mass events	Physical Activity	Martial arts dancing	Physical Activity

Attendance at zoo animal park botanic garden	Physical Activity	Martial arts dancing individual	Physical Activity
Babysitting	Physical Activity	Martial arts dancing unstructured	Physical Activity
Chess card paper and board games crosswords	Physical Activity	Motor sports roller sports cycling	Physical Activity
Chores	Physical Activity	Motor sports roller sports cycling individual	Physical Activity
Cleaning teeth	Physical Activity	Motor sports roller sports cycling unstructured	Physical Activity
Clothes making	Physical Activity	Organised individual sport and training other	Physical Activity
Clubs	Physical Activity	Organised team sports and training other	Physical Activity
Design home improvement	Physical Activity	Umpiring work	Physical Activity
Food drink clean up	Physical Activity	Unstructured active play other	Physical Activity
Food drink preparation	Physical Activity	Water ice snow sports	Physical Activity
Games of chance gambling	Physical Activity	Water ice snow sports individual	Physical Activity
Going out	Physical Activity	Water ice snow sports unstructured	Physical Activity
Handwork crafts excluding clothes making	Physical Activity	Sleeping napping not end of day bed time	Sleep
Heat water power upkeep	Physical Activity	Sleep	Sleep
Hobbies collections	Physical Activity		

Time-use diaries: Wave 3 Birth Cohort

In Wave 3, a parent/guardian was asked to complete a paper-based time-use diary for two days. The diary had a different format to the Wave 6 diary (Figure 2).

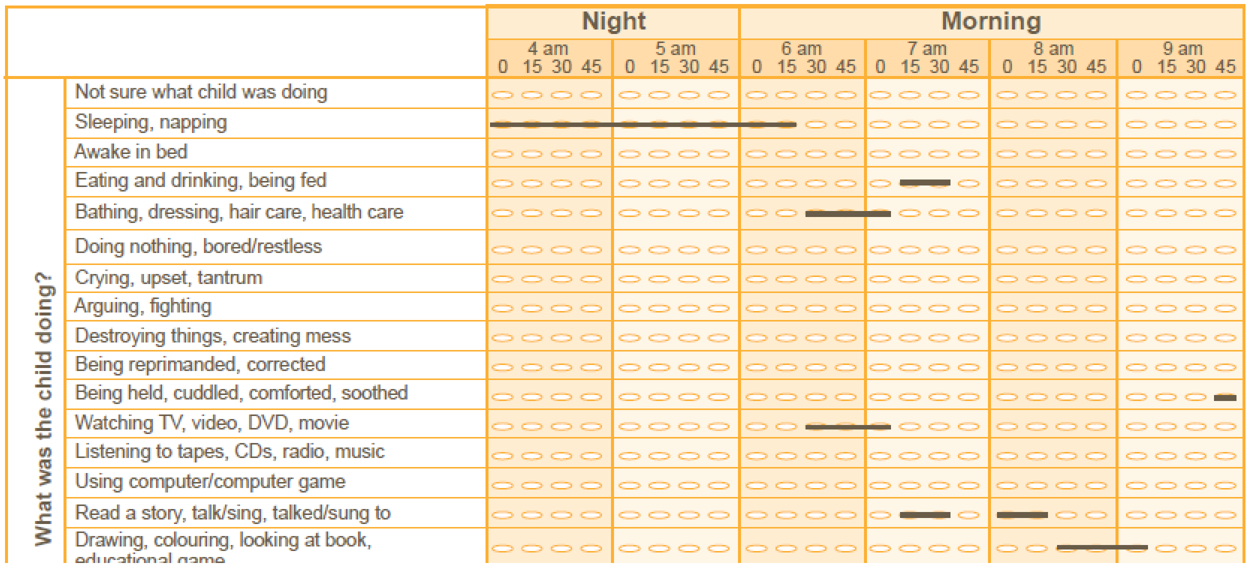

Figure 2. Example of a completed Wave 3 (Birth Cohort) time-use diary.

For the purposes of the main paper, we classified the activities into three categories – physical activity, sedentary behavior and sleep (Table 2), and averaged the time spent in these activities across the two recorded days.

Table 2. Classification of Wave 3 Birth Cohort Activity Codes into Physical Activity, Sedentary Behaviour and Sleep.

**Activity**	**Classification**
Not sure	NA
Sleeping, napping	Sleep
Awake in bed	Sedentary Behaviour
Doing nothing, bored restless	Sedentary Behaviour
Bathing, dressing, hair care, health care	Physical Activity
Eating and drinking, being fed	Sedentary Behaviour
Crying, upset, tantrum	Physical Activity
Destroying things, create mess	Physical Activity
Arguing, fighting	Physical Activity
Being reprimanded, corrected	Physical Activity
Held, cuddled, comforted soothed	Sedentary Behaviour
Watching TV, video or DVD	Sedentary Behaviour
Listening to tapes, CDs, radio, music	Sedentary Behaviour
Using computer/computer game	Sedentary Behaviour
Being read to, told a story, or sung to	Sedentary Behaviour
Drawing, colouring, looking at book, educational game	Physical Activity
Quiet free play (eg jigsaw, craft, dress-up)	Physical Activity
Active free play (running, climbing, ball game)	Physical Activity
Being taught to do chores, read etc	Physical Activity
Visiting people, special event, outing	Physical Activity

Organised lesson/activity	Physical Activity
Walk for travel or fun	Physical Activity
Travel in pusher of bike seat	Sedentary Behaviour
Travel in car	Sedentary Behaviour
Public transport	Sedentary Behaviour
Ride bike, trike etc (travel or fun)	Physical Activity

Anthropometric measurements in Wave 6

In the main paper, we explore the relationship between Wave 6 time use and body mass index (BMI) z-score. BMI was calculated as weight/height squared (kg/m^2). Children's weight in light clothing to the nearest 50g was measured by trained interviewers using glass bathroom scales. The interviewer measured child height twice, without shoes, to the nearest 0.1cm using a portable rigid stadiometer. The average of the two measurements was used; where they differed by more than 0.5cm, a third measurement was taken and the average of the closest two was used. The raw BMI were transformed using the 2000 US Centers for Disease Control (CDC) growth reference data [2]

Measurement of covariates

The participating child's exact age was obtained from their Medicare records, and their sex from parental survey. Socioeconomic position was captured using a previously constructed composite z-score which included parent-reported information about household income, parental education and parental employment [3].

Wave 6 participant characteristics

The analyses in the main paper included participants in the Wave 6 Birth cohort with complete and valid data for activity composition, BMI, sex, age and socioeconomic position (n=3228). Characteristics of included participants are shown in Table 3.

Characteristic		Summary statistics (n=3228)
Sex (n, %)	Male	1638 (51)
	Female	1590 (49)
Age (y) (mean (SD)		10.9 (0.3)
Socioeconomic position *z*-score		0.0 (1.0)
Body mass index *z*-score		0.3 (1.0)
Activity composition (%)	[Sleep; Sedentary; Physical Activity]	[43; 38; 19]

References

1. Corey, J.; Gallagher, J.; Davis, E.; Marquardt, M. *The times of their lives: Collecting time use data from children in the longitudinal study of australian children (lsac). Technical paper 13.*; Australian Bureau of Statistics, 2014.
2. Kuczmarksi, R.; Ogden, C.; Guo, S.; Grummer-Strawn, L.; Flegal, K.; Mei, Z.; al, e. 2000 cdc growth charts for the united states: Methods and development. *Vital and Health Statistics: Series 11. Data From the National Health Survey* **2002**, *246*, 1-190.
3. Blakemore, T.; Strazdins, L.; Gibbings, J. Measuring family socioeconomic position. *Australian Social Policy* **2009**, *8*, 121-168.
